# The Two-Systems Account of Theory of Mind: Testing the Links to Social- Perceptual and Cognitive Abilities

**DOI:** 10.3389/fnhum.2018.00025

**Published:** 2018-01-31

**Authors:** Bozana Meinhardt-Injac, Moritz M. Daum, Günter Meinhardt, Malte Persike

**Affiliations:** ^1^Department of Psychology, Johannes Gutenberg University Mainz, Mainz, Germany; ^2^Department of Psychology, University of Zurich, Zurich, Switzerland

**Keywords:** theory of mind, social perception, face recognition, language, individual differences

## Abstract

According to the two-systems account of theory of mind (ToM), understanding mental states of others involves both fast social-perceptual processes, as well as slower, reflexive cognitive operations (Frith and Frith, 2008; Apperly and Butterfill, 2009). To test the respective roles of specific abilities in either of these processes we administered 15 experimental procedures to a large sample of 343 participants, testing ability in face recognition and holistic perception, language, and reasoning. ToM was measured by a set of tasks requiring ability to track and to infer complex emotional and mental states of others from faces, eyes, spoken language, and prosody. We used structural equation modeling to test the relative strengths of a social-perceptual (face processing related) and reflexive-cognitive (language and reasoning related) path in predicting ToM ability. The two paths accounted for 58% of ToM variance, thus validating a general two-systems framework. Testing specific predictor paths revealed language and face recognition as strong and significant predictors of ToM. For reasoning, there were neither direct nor mediated effects, albeit reasoning was strongly associated with language. Holistic face perception also failed to show a direct link with ToM ability, while there was a mediated effect via face recognition. These results highlight the respective roles of face recognition and language for the social brain, and contribute closer empirical specification of the general two-systems account.

## Introduction

The ability to make sense of the behavior of others is fundamental for social interaction (Hampton et al., 2008; Slaughter et al., 2015). How humans deal with this challenging task is, however, still an unresolved question (Hasson and Frith, 2016). Premack and Woodruff (1978) first defined strategies for ascribing mental states to others (and to oneself) by the term “Theory of Mind” (henceforth ToM). Newer findings from research fields such as developmental psychology, social neuroscience, and research on disorders characterized by social deficits (e.g., autism, schizophrenia) showed that ToM is a complex construct comprising various processes (Mitchell, 2005; Kennedy and Adolphs, 2012; Schaafsma et al., 2015; Rice et al., 2016). Understanding and predicting behavior of others certainly involves attribution and/or inferring feelings, intentions, and beliefs from observable cues conveyed in human action, motion, and facial expression (Schaafsma et al., 2015).

Recently, a two-systems framework was propagated, which distinguishes implicit and explicit processes as the two major classes of processes involved in ToM (Keysers and Gazzola, 2007; Frith and Frith, 2008; Apperly and Butterfill, 2009). Socially relevant information is assumed to be transmitted by different signal systems, such as vocalization, facial expression, gaze direction, and body motion. Decoding of such socially relevant cues unfolds via implicit processes that are automatic, immediate, and reflex-like. However, humans also have knowledge and implicit theories on what kind of behavior and which reactions are expected in social situations – knowledge that is shaped by individual experience and by culture (Dunn et al., 1991; Shahaeian et al., 2011). The processes that are involved in explicit representation of the others’ mental states and beliefs are thought to be cognitively demanding, reflective and slow (Keysers and Gazzola, 2007; Frith and Frith, 2008; Apperly and Butterfill, 2009). While the integrative view on ToM processes outlined by the two-systems account seems to be compelling, comprehensive empirical evidence is still missing.

One potential reason for this is the complexity of involved implicit and explicit processes. Empirical evidence can only use single facets of either component and try to link them with ToM ability, which, again, is a complex construct spanning a wider range of abilities. In the present study, we adopted an individual differences approach to prove the relationship between social-perceptual and cognitive abilities as two basic components from either of the two systems, and ToM. To capture key aspects of ToM we operationally defined it as the ability to process complex and social emotional states from eyes, face, and voice, including meaning of spoken sentences and prosody cues. The reading the mind in the eyes test (henceforth, RME; Baron-Cohen et al., 2001) is one of the most frequently used tests of advanced ToM in clinical settings with groups with autism, Asperger’s syndrome, and schizophrenia (e.g., Baron-Cohen et al., 2001; Browne et al., 2016; Sato et al., 2017). The widespread use is not limited to populations with social deficits, but the test is also sensitive to cultural differences (e.g., Adams et al., 2010) and to individual differences of healthy individuals (e.g., Vellante et al., 2013; Preti et al., 2017). In this test, participants are required to attribute the mental state of a person shown in a photograph of the eyes region. While in the RME-test the stimuli are static, recognition of complex emotional mental states from dynamic faces, and, additionally, based on voice prosody and content of the spoken sentences is required in the Cambridge Mindreading (CAM) Face-Voice Battery (Golan et al., 2006). The test has been shown to discriminate between clinical (Autism, Asperger syndrome) and non-clinical groups (Golan et al., 2006) and it is sensitive to developmental changes in ToM (Vetter et al., 2013; Mahy et al., 2014). Recognition of complex emotional states is assumed to involve higher-level integration and mindreading, but also low-level perceptual processes (Mitchell and Phillips, 2015). Hence, there are good reasons to expect that both cognitive and perceptual processes are relevant for the ability to infer mental states in these selected ToM tasks.

As basic components of social-perceptual abilities, face perception and recognition could also be key proficiencies for ToM. The human face represents a wealth of social signals such as identity, gender, age, physical health, emotion, and intentions (Stein et al., 2014; Jack and Schyns, 2015). The question whether these different kinds of signals in faces are handled independently or interact with each other is so far unresolved. While some models postulate functionally and neurologically independent systems for processing facial identity, emotions, and facial speech (Haxby et al., 2000; Harris et al., 2014), other studies provide evidence that these processes are at least partly overlapping (Calder and Young, 2005; Fisher et al., 2016). Moreover, face identification and basic emotion recognition are both facilitated by *holistic* face processing mechanisms (Calder et al., 2000; Calder and Jansen, 2005; Tanaka et al., 2012). Holistic processing is viewed as an adaptive mechanism that arises through everyday expertise with faces and allows efficient and automatic processing of all relevant face information (Richler and Gauthier, 2014; Meinhardt-Injac et al., 2017). Studies adopting the individual differences approach have identified holistic processing as a valid predictor of individual differences in face recognition once the proper measures are chosen (Richler et al., 2011; DeGutis et al., 2013). Furthermore, face recognition alongside with fluid cognitive abilities (e.g., figural reasoning, working memory, immediate and delayed memory) have been shown to predict individual differences in basic emotion recognition (Hildebrandt et al., 2015). However, basic and complex social emotions involve partially different neuronal pathways (Burnett et al., 2009; Gilead et al., 2016) and recognition of complex social emotions may be less dependent on holistic processing than it is the case in basic emotions (Baron-Cohen, 2017). Experimental data supporting this conclusion are so far missing.

As outlined in the two-systems account (Apperly and Butterfill, 2009) explicit processes of mindreading are cognitively demanding, and heavily depend on language and reasoning ability. Only humans code and decode knowledge about the (social) world by means of symbolic language systems (Fitch et al., 2010). From a developmental perspective, language seems to be a critical aid for ToM development. Knowledge about the mental states of others and different aspects of language proficiency (e.g., general language, semantic, syntax) are strongly related during the course of development across childhood (for a review see Cutting and Dunn, 1999; Milligan et al., 2007). A tight relationship between language proficiency and ToM has been shown to persist in adulthood (Pyers and Senghas, 2009; Peterson and Miller, 2012). Some authors suggest that language *per se* is inextricably linked with representing and understanding mental and emotional states of others, as it entails the capacity for representation and reasoning (Barrett et al., 2007; Newton and de Villiers, 2007; Lupyan and Clark, 2015). While the relationship between language and ToM is well-established, it is less clear what the relationship between reasoning and ToM is. It seems that flexible reasoning in non-social situations and the ability to employ complex decision rules is a *necessary*, albeit not sufficient condition for ToM (Pruett et al., 2015). Although the results are somewhat inconsistent, there seems to be small, but significant positive correlation between individual differences in the RME test and individual differences in higher-reasoning abilities (for a review see, Baker et al., 2014). Moreover, reasoning and language are tightly interconnected and language is seen as being supportive of higher-order reasoning (Premack, 2004, 2007; Baldo et al., 2015).

To summarize, abilities of holistic face perception and recognition, as well as cognitive functions such as language and higher-order reasoning, either representing facets of the postulated two systems, could be potential drivers of ToM ability. In the present study we provide an empirical test of this theoretical framework, expecting that individual differences in ToM are predicted by individual differences in holistic face perception and face recognition as perceptual processed and by language and reasoning as cognitive processes.

## Materials and Methods

### Study Outline, Methodological Approach, and Predictions

Holistic face perception (HP), face recognition (FR), relational reasoning (RE) and language (LA) are complex abilities, requiring multiple empirical indicators for their valid representation. We postulate that ToM is predicted by these four domains of ability, which means that we have a directed main hypothesis. The method of choice in this situation is to represent the five abilities as latent factors, to be derived from multiple indicators, and to link the factors in directed paths using linear structural equation modeling (SEM; Bollen, 1989). This approach allows us to generalize across experimental paradigms and measurement error of single tests, while it enables testing hypotheses about directed (causal) effects among latent constructs.

In the framework of SEM, both the adequacy of the representation of measures by latent factors (“measurement model”) and the significance and the degree of explained variance in the to-be-explained (endogeneous) constructs can be statistically tested and evaluated. It is important to note that these are independent steps of SEM. Once confirmed the adequacy of the measurement model by methods of confirmatory factor analysis, the outcome of the structural modeling depends solely on the correlation structure among the latent factors. If there are no substantial correlations, also directed path modeling, which is a theory-driven proposal for reproducing these correlations, fails.

Based on reasoning outlined in the Introduction, we consider the following predictions:

[**P1**] *ToM shows significant correlations with HP, FR, RE, and LA.*
*Comment.* Based on existing evidence (s.a.) we expect correlations of ToM with abstract reasoning to be somewhat smaller than with language and/or face processing related ability.
[**P2**] *ToM is predicted by HP, FR, RE, and LA.*
[**P3**] *RE is predicted by LA.*
[**P4**] *FR is predicted by HP.*
*Comment.* SEM should prove a significant direct path from HP to FR if proper measures of HP are used (DeGutis et al., 2013).
Note that, since P3 and P4 postulate direct paths HP→FR and LA→RE, indirect causal (mediated) effects of HP→ToM via FR and LA→ToM via RE are implied by the set of predictions P2–P4.

At the current state of empirical findings we cannot be more specific about the relative strengths of the explanatory paths. A strong link of ToM and language is well-established (s.a.). Confirmation of this link would thus validate our measurement models of ToM and LA. Since this is the first attempt to testing a potential link of face processing abilities to ToM, we cannot be more specific about its relative strength compared to the link with LA, albeit we postulate this link theoretically.

### Participants

A sample of 343 participants (age between 17 and 40 years; *M* = 22.23, *SD* = 3.38; 71% female) was tested. The majority of the participants in the sample (290 out of 343) were between 18 and 25 years of age. More than 92%, of participants were students of the university of Mainz in various disciplines, the remainder 8% came from different professions, mostly from administration and service. Data collection was conducted in two experimental sessions, each lasting about 1 h, including breaks. We recruited via university information material (in e-mails and on flyers) and participants received monetary compensation for participation. None of the participants reported impairments in perception, hearing, or cognitive functions. All subjects reported no serious head injuries.

### Materials and Procedure

**FIGURE 1:**
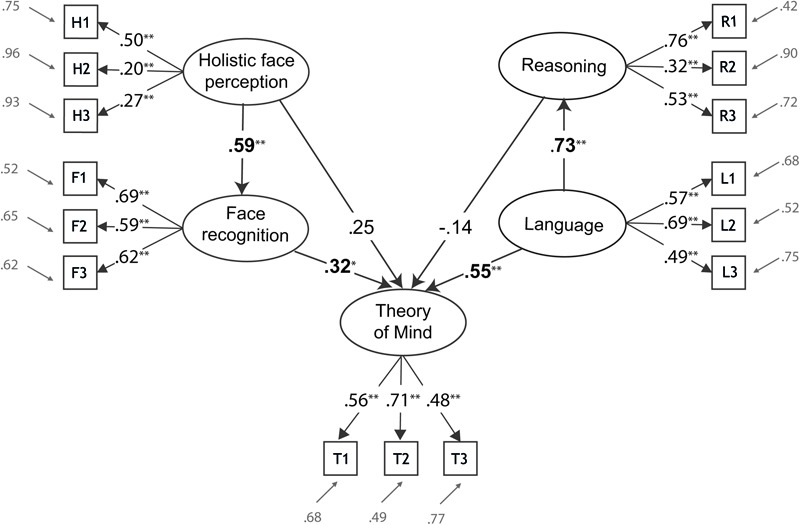
Complete SEM model (measurement and structural model) for predicting ToM by language, reasoning, holistic face perception and face recognition ability. Empirical indicators are depicted as rectangles and have alphanumeric codes (see section “Materials and Methods”). Latent factors are displayed as ellipses. Standardized path coefficients are shown with their path arrows. Significant path coefficients are marked by (^∗^*p* < 0.05) and (^∗∗^*p* < 0.01), and are printed boldface. Residual variances for all indicators are shown in gray.

In what follows, the tasks used to measure the different abilities are described. Each task contributed one or two aggregate scores (indicators), which we used to derive the respective latent factors. To facilitate readability, alphanumeric codes identify indicators throughout the text as well as in **Table 1** and **Figure 1**.

**Table 1:** Descriptive statistics of all measurement variables.

Face recognition	*M*	*SD*	*M′*	*SD′*	*Indicator code*
CFMT-upr	0.73	0.14			F1
CFMT-inv	0.57	0.10			F2
GFMT	0.81	0.11			F3
**Holistic face perception**					
Composite Task-CC	0.87	0.09	0.00	0.09	H1
Composite Task-IC	0.72	0.11			
Context Congruency Task_CC-upr	0.88	0.12	0.00	0.10	H2
Context Congruency Task_CC-inv	0.77	0.12			
Context Congruency Task_IC-upr	0.64	0.14	0.00	0.12	H3
Context Congruency Task_IC-inv	0.59	0.13			
**Language**					
Vocabulary-MWTB	0.77	0.10			L1
Verbal Intelligence	0.54	0.13			L2
Orthography	0.40	0.14			L3
**Reasoning**					
Abstract reasoning–Raven Test	0.73	0.13			R1
Figural Intelligence	0.74	0.21			R2
Digit Sequence Completion Task	0.64	0.21			R3
**Theory of mind (ToM)**					
Reading the Mind in the Eyes (RME)	0.75	0.09			T1
Cambridge Mindreading Face Battery	0.72	0.08			T2
Cambridge Mindreading Voice Battery	0.64	0.09			T3

*Sample size was *N* = 343. The Table shows mean (M), standard deviation (SD) of the original scales, mean and standard deviation of indicator values derived from the original scales for the holistic face perception measures (M′ and SD′), and indicator code.*

We used three indicators of face recognition ability to estimate a face recognition factor (F1, F2, and F3). Two stem from the Cambridge Face Recognition Test and one from the Glasgow Face Matching Test. Face perception ability was measured by three indicators of holistic face perception (H1, H2, and H3). The latent factor representing language ability was established based on three indicators from three tasks measuring vocabulary, verbal analogies and orthography (L1, L2, and L3). The latent factor for the reasoning ability was established based on the indicators gained in the short version of the Raven Test (R1), and two subtests from IST-2000 (Liepmann et al., 2007), which tests figural intelligence (R2, dice task) and numerical intelligence (R3, digit sequence completion task). Indicators from three tasks (T1, T2, and T3) requiring recognition of complex mental states from the eyes (Reading the Mind in the Eyes Test), from videos (Cambridge Mindreading Face Battery) and from voice (Cambridge Mindreading Voice Battery) were used to establish a measurement model for ToM. A detailed description of all indicators, including indicator codes, is given below.

#### Face Recognition (FR)

##### [F1 and F2] Cambridge Face Memory Test (CFMT)

The Cambridge Face Memory Test (Duchaine and Nakayama, 2006) was developed to study briefly delayed face identity recognition, covering generalization across viewpoint and image distortion. The test comprises six target identities and 46 distractor identities. There are two versions of the test, one with upright and one with inverted face stimuli, each encompassing 72 trials. The proportion of correct responses in upright and inverted versions of the test was measured. The CMFT is freely available from the authors for scientific purposes.

##### [F3] Glasgow Face Matching Test (GFMT)

The Glasgow Face Matching Test (GFMT) uses photos of same or different people, taken in similar lighting and pose, but with two different cameras, which allows for testing identity-to-identity rather than picture-to-picture matching. Here the short version of the GFMT, with a test reliability of 0.91 (see Burton et al., 2010) was used. The test comprised 40 face pairs, 20 showing same and 20 showing different persons.

#### Holistic Face Perception (HP)

##### [H1] Composite Paradigm (CC/IC)

We used the complete composite paradigm (Gauthier and Bukach, 2007). Composite stimuli were created by combining the top-half of one face with the bottom-half of a second face. Subjects were asked to decide whether either the upper or the lower face halves in two successively shown composite face images were the same or different. In the congruent condition (CC) the identity relation of attended and unattended face halves was the same in a pair of presented faces. In the incongruent condition (IC) identities of the non-attended face halves differed from the identities of the attended face halves. As indicator of holistic processing (H1) the residual regression scores were calculated as the CC performance not accounted for by IC performance at the level of individual data.

##### [H2 and H3] Context Congruency Paradigm (CC/IC)

The context congruency paradigm (Meinhardt-Injac et al., 2010) measures holistic processing of external (hair, ears, shape) and internal (eyes, eyebrows, mouth, nose) features in a face. Subjects were asked to decide whether internal features of two successively presented composite faces were the same or different. Congruent and incongruent trials were constructed by following the same logic as in the composite paradigm. Stimuli were presented randomized in upright and inverted orientation. The indicator of holistic processing was calculated as the residual performance in upright orientation that was not accounted for by performance in inverted orientation, both for congruent (H2) and incongruent (H3) trials. For more details on using regression to measure holistic processing, see DeGutis et al. (2013).

#### Reasoning (RE)

##### [R1] Raven Test

A short version of Raven’s standard progressive matrices task (Raven, 2000) was used to measure abstract reasoning. In 40 trials, listed in order of difficulty, participants were asked to identify the missing element, which completes a given pattern. All trials had a visual geometric design with a missing piece. Subjects were asked to choose one out of eight elements to complete the matrix.

##### [R2] Figural Intelligence

An adapted version of the IST-2000 dice task (Liepmann et al., 2007) was used to measure visual thinking abilities. Participants were asked to pick the only die from the five dice that depicted all spatial features as seen in the cue die. The other four dice showed small differences in featural organization, or the organization of all three sides was modified. Twelve trials were presented.

##### [R3] Digit Sequence Completion Task

A short version of the IST-2000R (Liepmann et al., 2007) digit sequence completion task was conducted to measure logical thinking abilities. In 40 trials, listed in order of difficulty, a sequence of seven digits was presented. Participants were asked to complete the sequence logically and choose the finishing digit out of the given four digits.

#### Language (LA)

##### [L1] Vocabulary Test MWT-B

The MWT-B was used to estimate the treasury of words. In 37 trials, participants were asked to pick the only real word from a five-word sequence. The target word was the only real word in a sequence of artificial distracter words. Detailed information on test construction can be found in Lehrl (2005).

##### [L2] Verbal Intelligence

A short version of the IST-2000R (Liepmann et al., 2007) analogy task was used. Per trial, three cue words were presented. The first two words were connected via a particular semantic relation. The third cue word had no relational word (e.g., “to breathe: lung = to sweat: ?”). Along with the cue words, five other words were presented. One of them (the target word) had a similar relation to the third cue word. The other four words (e.g., sun, effort, sweat, temperature) were distractors. Participants were asked to identify the matching word from the five presented words. The test comprised 40 trials, listed in order of difficulty.

##### [L3] Orthography Test

The test measures orthography and grammar knowledge. Two different kinds of trials were used. In Trial 1, four almost identical German sentences were presented, with three of them comprising only petite differences in spelling, punctuation or in the use of capital or small initial letters (distractors), and one of them being the only orthographically and grammatically correct sentence (target sentence). Participants were asked to read every sentence thoroughly and to find the target sentence. In Trial 2, three of the presented sentences were orthographically and grammatically correct (distractors) and one sentence included a mistake. Herein, participants were asked to identify the only incorrect sentence. Prior to each of the 17 trials the subjects received the instruction to identify the only correct or the only incorrect sentence.

#### Theory of Mind (ToM)

##### [T1] Reading the Mind in the Eyes

This test measures the ability to understand complex mental states from cues contained in the eyes region of a human face (Baron-Cohen et al., 2001). Grayscale photographs of the eyes region of different actors, each revealing a complex emotional or mental state, were presented to participants consecutively. With each photo, four adjective descriptions of complex emotions or mental states were presented, one of them matching the expression shown in the photo. Participants were asked to choose the adjective matching the expression from the present photo best. There were 36 trials in total.

##### [T2 and T3] Cambridge Mindreading (CAM) Face-Voice Battery

CAM-F and CAM-V are two separate subtests, whereby either face or voice stimuli are used for recognition of complex mental and emotional states (Golan et al., 2006). In the CAM-F, participants were shown 3–5 s videos of actors portraying one out of 20 complex emotion concepts. There were 50 videos shown in total. Subjects were asked to decide which of the four presented adjectives matched the expressed emotion from the video best.

For the CAM-V, participants were asked to put headphones on. Thereafter, they were presented 50 individual sentences, spoken in a particular emotional intonation, each representing one out of the 20 complex emotion concepts also used in the video task. After subjects listened to a sentence, four adjective descriptions of emotions were presented, one of them matching the vocal expression from the voice recording. Again, subjects were asked to match the emotion from the voice recording to the adjective that fitted best. Like in the video task, overall emotion perception from vocalization was the main outcome, measured as the sum all correctly identified emotion expressions.

#### Indicator Scores

**Table 1** shows the basic statistics of the indicator scores, which were proportions of correct responses within the module specific tasks, except for the indicators of holistic face perception, H1, H2, and H3. Based on recommendations of DeGutis et al. (2013), we calculated residuals from regression equations relating the score of interest (*y-*variable) to the score in conditions where holistic processing is expected to be absent, or reduced (*x*-variable). Before regression residuals were calculated it was ascertained that the expected experimental effects existed, thus verifying the main prerequisite of the regression method. Analyzing the data of the composite face paradigm showed a large congruency effect (Δ = 0.16, s_e_ = 0.006, *t* = 25.9, *p* < 0.001, Cohen’s *d* = 1.40). For the context congruency paradigm we also found a large congruency effect (Δ = 0.24, s_e_ = 0.005, *t* = 27.1, *p* < 0.001, *d* = 1.46), which was attenuated when faces were inverted (Δ = 0.18, s_e_ = 0.008, *t* = 21.4, *p* < 0.001, *d* = 1.16). The effect of orientation was large in congruent, but smaller in incongruent condition (congruent: Δ = 0.11, s_e_ = 0.006, *t* = 19.4, *p* < 0.001, *d* = 1.06; incongruent: Δ = 0.05, s_e_ = 0.007, *t* = 7.0, *p* < 0.001, *d* = 0.38).

### Analysis Regime

As the first step in SEM the measurement model for the latent constructs is defined. Consistently for each of the five constructs we used three indicators, explained by a single latent factor. Since confirmatory factor analysis pursues to adequately reproduce the covariance structure among the indicators, but not necessarily their actual values, adequacy of the measurement model is assumed if covariance matrix **C** of observed indicators and covariance matrix **C′** of the indicators predicted by the measurement model coincide. This is usually tested by evaluating deviations with a χ^2^ statistic (Bollen and Long, 1993). Because the test is rather sensitive to hurt multivariate normality assumption, additional fit indices are considered to evaluate the model fit (see Hu and Bentler, 1999). Among various fit indices, we adopted the commonly used: root-mean square error of approximation (RMSEA), and comparative fit index (CFI). CFI values of 0.95 or higher indicate excellent model fit, but values below 0.90 indicate weak or lacking fit and lead to the rejection of the model. RMSEA values in the range of 0.05 to 0.08 indicate acceptable fit, while higher values indicate unacceptable fit. RMSEA values below 0.05 are considered as indicating good or very good model fit (see Hu and Bentler, 1999, for details). Further tests of the adequacy of the measurement model concern the deviation of observed correlation matrix **R** and model correlation matrix **R′** (correlation residuals). Since the structural model comprises the regression equations for each endogeneous variable, it is evaluated by testing its path coefficients (standardized regression coefficients) for significance, and by evaluating the proportion of explained variance of each equation. We performed SEM with the Mplus statistical package (Muthén and Muthén, 2010). Maximum-likelihood parameter estimates were used with no constraints for path coefficients or correlations. For convenience, latent variable variances were fixed to 1. In total, 50 parameters were estimated. With a sample size of *N* = 343, this amounts to 6.9 subjects per estimated parameter, which is still considered as a favorable ratio in the SEM literature (Schreiber, 2008).

## Results

### Adequacy of Measurement Model

The χ^2^ test for agreement of observed and model covariance matrix indicated deviation (χ^2^ = 116.3, *df* = 83, *p* < 0.01). However, comparative fit index (CFI = 0.952) as well as RMSEA and its confidence interval (RMSEA = 0.034, 90% CI for RMSEA = [0.018, 0.048]) both indicated good or very good model fit, respectively. Such results patterns with conflicting results from the χ^2^ test and alternative fit indices are frequent in SEM studies (see, e.g., Hildebrandt et al., 2015). A potential reason for the significance of the χ^2^ test could lie in deviations in the variance structure. We therefore tested whether there were any significant residual correlations in **R_e_** = **R**–**R′**, which would indicate failure to reproduce the indicator correlations by the measurement model. Reviewing the 105 residual correlations showed that their maximum value was still not significant (*r*_e_ = 0.046, *t* = 0.85, *p* = 0.397), confirming to us that the measurement model was apt to reproduce the empirical correlation structure of the 15 indicators with good accuracy. Together with the results for the alternative fit indices, testing results for the measurement model thus indicated that the latent factors adequately represented the test indicators.

### Structural Model

In SEM the path equations are solved by decomposing the correlations among the latent variables (see **Appendix**). **Table 2** shows the correlations. In agreement with our first prediction (P1), ToM showed high bivariate correlations of about 0.55 with HP, FR and LA, while the correlation with RE was lower. The correlation of HP and FR was in the same order of magnitude, slightly exceeding the value reported by DeGutis et al. (2013), who found *r* = [0.36, 0.46], depending on the test used. The highest correlation, *r* = 0.73, was found between LA and RE, while RE did just modestly correlate with HP and FR.

**Table 2:** Estimated latent factor correlations.

	ToM	HP	FR	LA	RE
ToM	1.00				
HP	0.56	1.00			
FR	0.54	0.59	1.00		
LA	0.57	0.26	0.16	1.00	
RE	0.35	0.19	0.11	0.73	1.00

*The critical correlations for a given significance level are *r*(0.05) = 0.11, *r*(0.01) = 0.14, and *r*(0.001) = 0.18.*

Our prediction P2 postulated directed paths to ToM in the structural model (see connected ellipses in **Figure 1**). Significant path coefficients are printed boldface. We found two significant direct paths, LA→ToM and FR→ToM, while the direct paths from HP and RE failed to reach significance. However, the multiple correlation for explaining ToM by the four predictors was *R* = 0.76, which amounts to 58% of explained variance (*R*^2^ = 0.58, s_e_ = 0.102, *t* = 5.65, *p* < 0.001). This suggests potential mediated (indirect) effects for explaining ToM (see below).

P3 and P4 were confirmed by the significant and strong path coefficients, which coincided with their bivariate correlations, since there was only one predictor in their regression equations. FR was explained by HP with a 35% variance proportion (*R*^2^ = 0.35, s_e_ = 0.14, *t* = 2.5, *p* < 0.01) and LA explained 53% of RE variance (*R*^2^ = 0.53, s_e_ = 0.11, *t* = 5.0, *p* < 0.001).

The bivariate correlation of ToM and HP was *r* (ToM-HP) = 0.56, but HP received a much lower path coefficient of 0.25 in the direct HP→ToM path. Since this coefficient reflects the effect of HP on ToM controlled for FR, this indicated a mediated effect of HP via FR. The maximum-likelihood estimate of the mediated effect was 0.19, which practically coincided with the product of the path coefficients. Testing for significance with Sobel’s test (Sobel, 1982), which is a relatively conservative test of mediation (Fritz and Mackinnon, 2007) showed marginal significance (*z* = 1.82, *p* < 0.068). Other authors (MacKinnon et al., 2002) consider mediation to be present if both path coefficients of the indirect path are significant, which was the case for the HP→FR→ToM path (see **Figure 1**).

Things were different in the paths involving LA and RE. In the direct LA→ToM path the coefficient for LA was 0.55, which was very close to its correlation with ToM. Since this path coefficient describes the effect of LA on ToM controlled for RE, this already indicated that there was no further leeway for a mediated effect of LA via RE. The path coefficient relating RE to ToM was small and non-significant, limiting the estimate for the indirect effect to -0.1. Accordingly, Sobel’s test indicated clear non-significant results (*z* = -0.86, *p* = 0.392).

Taken together, the structural modeling results revealed different structures in the ToM paths coming from HP and FR on the one hand and from LA and RE on the other. Albeit statistically significant, RE had clearly the lowest criterion correlation of all four ToM predictors. Structural modeling revealed a strong direct effect of language on ToM, while direct and indirect effects via reasoning were absent. In the ToM paths coming from HP and FR, criterion correlations and predictor intercorrelations were high, and at equal strengths. Structural modeling showed effects on ToM from both predictors, but emphasized face recognition over holistic face perception, which exerted only a mediated effect on ToM via face recognition.

## Discussion

The human ability to track and infer mental states of others (i.e., Theory of Mind) likely involves different perceptual and cognitive processes (Mitchell, 2005; Schaafsma et al., 2015), as outlined in the framework of the two-systems account. These processes fall into implicit processes that are automatic, reflex-like and efficient, and slow, cognitively demanding and reflective explicit processes (Keysers and Gazzola, 2007; Frith and Frith, 2008; Apperly and Butterfill, 2009). Implicit processes comprise decoding of socially relevant information transferred by facial expression, voice, and body motion. The explicit representation of the others’ mental states should involve cognitive skills, such as language and higher-order reasoning. Albeit a long-standing debate, yet no comprehensive empirical evidence has been provided in support of or against such a two-systems account.

In the present study we supplied comprehensive empirical evidence, gained from a battery of 15 tests administered to a large sample of 343 participants. Our results confirm the particular role of language, being an important facet of the slower reflexive processes, and reveal, for the first time, the strong contribution of face processing related ability as a relevant facet of fast and implicit processes, while they failed to show a particular relevance of reasoning ability. Thus, only perceptual and cognitive processes directly involved in processing of socially relevant information proved to predict the ability to infer complex emotional and mental states of others from observable cues. These results contribute closer empirical specification of a general two-stems framework (Keysers and Gazzola, 2007; Frith and Frith, 2008; Apperly and Butterfill, 2009; Schaafsma et al., 2015) and highlight the respective role of face processing and language ability for the social brain (Kennedy and Adolphs, 2012).

Our findings prioritize the role of face recognition over holistic perception for predicting ToM ability, which suggests a particular role of face-identity processing. This is supported by a study of Palermo et al. (2013), who found a substantial correlation of emotion matching and face recognition ability, measured by the CFMT. Using a larger sample of 269 subjects, Hildebrandt et al. (2015) found evidence for a link between face identity processing and basic emotion recognition, which coexisted with a link to general cognitive ability. Schweinberger and Soukup (1998) also found evidence for interdependence of face-identity and facial expression processing, showing that facial speech was recognized better when also face identity processing succeeded. These results indicate that ability to process personal identity and reading facial and emotional expression are closely related. These results correspond to findings showing that the ability to make mental state inferences form faces and social stories could be related to other processes of person perception, such as perception of body motion (Phillips et al., 2011; Rice et al., 2016).

Our structural modeling did not confirm a direct link of holistic face processing ability to ToM measures. While we found a direct link between holistic perception and face recognition, in line with previous findings (Richler et al., 2011; DeGutis et al., 2013), our failure to confirm a direct link between holistic processing and ToM measures is in contrast to studies indicating involvement of holistic processing in basic emotion recognition (Calder et al., 2000; Calder and Jansen, 2005; Tanaka et al., 2012). However, recognition of complex emotional and mental states from faces seem to be less dependent on face context than on the eyes region alone (Baron-Cohen, 2017). This could be a potential reason for the failure to find a strong direct link between holistic processing and ToM. Moreover, holistic face processing is not only a highly experience-dependent ability (Gauthier and Bukach, 2007), but also a perceptual strategy, that can be adopted or not, depending on the requirements of the task (Fitousi, 2016). Face recognition ability, on the other hand, is a basic ability (Wilmer et al., 2010), which cannot be applied or not, contingently with the situation. Our results *may* underestimate the role of holistic processes for face-based ToM. However, an experimentally oriented approach is necessary to elucidate role and use of holistic strategies in processing of complex emotional and mental states from visual cues in faces, as it was required in two out of three of our ToM tasks.

The strongest predictor of the individual differences in ToM in our model was language, represented as a latent factor from three different tasks that measured vocabulary, verbal intelligence, and orthography and grammar knowledge. The role of language skills for the development of ToM has been demonstrated in normally developing children (for a review see Milligan et al., 2007), but also in children with delayed language development (Nilsson and López, 2016), as well as in individuals with sensory deficits (Pyers and Senghas, 2009). In a study with adolescent and adult deaf learners of Nicaraguan Sign Language, Pyers and Senghas (2009) have demonstrated that the ability to reason about false beliefs followed the acquisition of more advanced language. The relationship between ToM and language has been found also in healthy adults when mental states are inferred from stories (Ahmed and Miller, 2011) and in RME Test (Peterson and Miller, 2012). However, strong predictive power of language for ToM task in the present study may, at least partly, reflect focus on verbal response options (Johnston et al., 2008). Despite limitations of single ToM tasks, language skills are seen as a necessary condition for ToM to develop (Pyers and Senghas, 2009) and as inextricably linked with representing and understanding mental and emotional states of others (Barrett et al., 2007; Newton and de Villiers, 2007; Lupyan and Clark, 2015). Taken together, previous and present findings suggest that language and ToM ability are strongly linked, not only across development, but also in adulthood.

In contrast to the previous findings (see Baker et al., 2014 for a meta-analysis), our results show that reasoning ability in non-social situations and the use of complex rules do not account for individual differences in ToM in healthy young adults. The relationship between ToM and reasoning is, however, more complex, since cognitive abilities may be considered as a necessary, but not sufficient prerequisite for ToM (Pruett et al., 2015). For example, in atypical development, mental disability below the normal range of intelligence impairs ToM performance in Down syndrome (Zelazo et al., 1996), but a well-developed reasoning ability in autistic persons is no sufficient condition to pass ToM tasks (Baron-Cohen et al., 1997). In persons with Williams syndrome language processing, face recognition and ToM are functional despite several deficits in general cognitive abilities (Karmiloff-Smith et al., 1995). These findings suggest that the impact of general cognitive ability on ToM might be modulated by language. Here, the modest correlation of reasoning and ToM did not give rise for a mediated effect in this direction. For further research on healthy individuals it would be likely more critical to include tests of executive functions rather than relational reasoning. Alongside with language, executive functions seem constantly related to development of ToM in childhood and adolescence (Dumontheil et al., 2010; Devine and Hughes, 2014; Hughes and Devine, 2015), and seem to remain a relevant predictor of the ToM ability in adulthood (Apperly et al., 2009; Ahmed and Miller, 2011).

We also found significant, albeit modest, correlation between holistic face processing and language. The holistic mechanism tapped with facial stimuli seems to reflect basic perceptual processes that are relevant not only when recognizing facial identity, but also when reading written words. The effect can be traced back to holistic effects in word processing (Wong et al., 2011). Indeed, extensive expertise in processing these stimuli leads to similar brain specialization in the neighboring areas – fusiform face area for faces (FFA; McGugin et al., 2012) and in visual word form area for written words (Baker et al., 2007). Common for faces and words, despite the obvious difference in appearance, is extensive experience that humans gather with these kinds of stimuli in everyday life, as well as their crucial role in human communication and social interactions.

## Conclusion

Our results show that social-perceptual and cognitive processes involved in the representation of socially relevant information are significant predictors of individual differences in the ability to track and to infer complex emotional and mental states of others from observable cues, including faces, eyes, spoken language and prosody. This in line with conclusions gained in studies on social cognition in psychiatric and neurological disorders (Karmiloff-Smith et al., 1995; Kennedy and Adolphs, 2012). Extensive experience and social relevance may drive specialization in each of these skills during development, resulting in longstanding individual differences.

## Ethics Statement

The research reported in this manuscript fully complied with the principles of the Declaration of Helsinki. We informed in written form about the study aims, methods, sources of funding, any possible conflicts of interest, and institutional affiliations of the researchers, and obtained written informed consent from all participants. They were free to abstain from participation or to withdraw consent to participate at any time without consequences. The data were analyzed anonymously. The local ethics committee of Johannes Gutenberg University Mainz approved all experimental procedures.

## Author Contributions

Authors contributed equally to the conceptualization of the study. BM-I and MP set up the basic design, and conducted experiments. BM-I, MP, and GM contributed data analysis and modeling. All authors were involved in writing, preparation of the manuscript and its final approval. All authors agree to be accountable for all aspects of the work, ensuring that questions related to the accuracy or integrity of any part of the work were investigated and resolved appropriately.

## Conflict of Interest Statement

The authors declare that the research was conducted in the absence of any commercial or financial relationships that could be construed as a potential conflict of interest.

## Appendix

According to the fundamental corollary of path analysis, path coefficients derive from estimated latent factor correlations,

(1) $$r_{cj}=\sum_{i}b_{ci}r_{ij}$$

(Wright, 1934), whereby variables *c* and *j* are directly linked with an arrow from *j* to *c*, and *i* runs over all exogeneous variables (origins of arrows). Now, setting *z*_5_ = ToM, *z*_1_ = HP, *z*_2_ = FR, *z*_3_ = RE, *z*_4_ = LA, and in view of the structural diagram shown in **Figure 1**, Eq. (1) generates the following equations for explaining ToM:

(2) $$\begin{array}{l} r_{51}=b_{51}r_{11}+b_{52}r_{21}+b_{53}r_{31}+b_{54}r_{41}\\ r_{52}=b_{51}r_{12}+b_{52}r_{22}+b_{53}r_{32}+b_{54}r_{42}\\ r_{53}=b_{51}r_{13}+b_{52}r_{23}+b_{53}r_{33}+b_{54}r_{43}\\ r_{54}=b_{51}r_{14}+b_{52}r_{24}+b_{53}r_{34}+b_{54}r_{44} \end{array}$$

which reads **r** = **Rb**, with **r** the vector of correlations of criterion *z*_5_ with the four predictors, **b** the vector of path coefficients for all paths to criterion *z*_5_ and **R** the (4 × 4) predictor correlation matrix. The system (2) is solved by finding the inverse of **R**, which exists if all predictors are linear independent, i.e., **b** = **R^-1^r**. With our ML estimates for the latent factor correlations (see **Table 2**) we find

$$r=\left(\begin{array}{l} 0.56\\ 0.54\\ 0.35\\ 0.57 \end{array}\right)R=\left(\begin{array}{l} 1.000.590.190.26\\ 0.591.000.110.16\\ 0.190.111.000.73\\ 0.260.160.731.00 \end{array}\right)b=\left(\begin{array}{l} 0.25\\ 0.32\\ -0.14\\ 0.55 \end{array}\right)$$

for the path coefficients to ToM. Since FR and RE receive only a single arrow, applying (1) shows that their path coefficients are *b*_21_ = *r*_21_ = 0.59 and *b*_34_ = *r*_34_ = 0.73, respectively.
